# Non-COVID activity in French emergency department during COVID-19 pandemic (March 2020 to March 2022)

**DOI:** 10.1093/eurpub/ckac131.054

**Published:** 2022-10-25

**Authors:** A Fouillet, C Forgeot, I Pontais, G Pedrono, C Caserio-Schönemann

**Affiliations:** Division of Data Sciences, Santé Publique France, Saint-Maurice, France

## Abstract

**Background:**

The COVID-19 epidemic and mitigation actions had major impacts on health and healthcare use by the French population. Since 2004, the French public health agency daily collects individual data of visits in the emergency departments (ED) OSCOUR® network (94% of national visits in 2022). We aimed to analyse the evolution of non-Covid ED visits from 2020 to March 2022, in order to identify potential indirect impact of the epidemic.

**Methods:**

The main medical diagnosis (MD) coded in ICD-10 from each ED visit from 2017 to March 2022 was classified into 17 ICD-10 chapters and in 95 disjoint subgroups of pathologies. The observed numbers of ED visits by age group and by chapters/subgroups were compared to expected numbers, estimated using an overdispersed Poisson regression model based on 2017-2019 data.

**Results:**

The observed numbers of ED visits for all chapters and for a large part of subgroups were significantly lower than the expected numbers during the three lockdowns in all age groups and progressively returned to the expected level in 2021. A change in the pattern of a limited list of subgroups was observed: ED visits for purpura, chronic blood diseases and neurologic disorders in children decreased during the first lockdown and remained under the expected level until March 2022. Inversely the number of ED visits for mental health and wheezing in children, for pulmonary embolism in adults and for neoplams in the elderly increased and remained over the expected values until 2022.

**Conclusions:**

Syndromic ED system was a pillar of the French reactive surveillance of direct and indirect impacts of COVID-19 epidemic. The changes observed for different subgroups of pathologies may reflect a negative impact of the epidemic, a positive effect of protective measures on the spread of other infectious diseases, a modification in the organization or in the use of health care in specific domains. Further studies using hospitalization data could explore these hypotheses.

**Key messages:**

• Existing syndromic surveillance system implemented before the emergence of SARS-COV2 enabled to monitor non-Covid-19 visits to emergency departments and assess changes in patterns of pathologies.

• An increase in the number of emergency department visits during the COVID-19 epidemic was observed for mental health in children, for pulmonary embolism in adults and for neoplams in the elderly.

